# Misinformation Drives Low Human Papillomavirus Vaccination Coverage in South African Girls Attending Private Schools

**DOI:** 10.3389/fpubh.2021.598625

**Published:** 2021-02-19

**Authors:** Tracy Milondzo, Johanna C. Meyer, Carine Dochez, Rosemary J. Burnett

**Affiliations:** ^1^Department of Public Health, University of Limpopo, Polokwane, South Africa; ^2^Division of Public Health Pharmacy and Management, Sefako Makgatho Health Sciences University, Pretoria, South Africa; ^3^South African Vaccination and Immunisation Centre, Sefako Makgatho Health Sciences University, Pretoria, South Africa; ^4^Network for Education and Support in Immunisation, Department of Family Medicine and Population Health, University of Antwerp, Antwerp, Belgium; ^5^Department of Virology, Sefako Makgatho Health Sciences University, Pretoria, South Africa

**Keywords:** human papillomavirus vaccine, vaccination coverage, South Africa, private sector schools, knowledge and attitudes, vaccine hesitancy, vaccine confidence, vaccine misinformation

## Abstract

**Background:** Cervical cancer, caused by persistent human papillomavirus (HPV) infection, is the leading cause of female cancer deaths in South Africa. In 2014, the South African National Department of Health introduced a free public sector school-based HPV vaccination programme, targeting grade 4 girls aged ≥9 years. However, private sector school girls receive HPV vaccination through their healthcare providers at cost. This study investigated HPV vaccination knowledge, attitudes and practices of caregivers of girls aged ≥9 years in grades 4–7 attending South African private schools.

**Methods:** A link to an online survey was circulated to caregivers via an email sent to school principals of all private schools in four provinces enrolling girls in grades 4–7. Following a poor post-reminder response, a paid Facebook survey-linked advert targeting South African Facebook users aged ≥25 years nationally was run for 4 days, and placed on the South African Vaccination and Immunisation Centre's Facebook page for 20 days.

**Results:** Of 615 respondents, 413 provided HPV vaccination data and 455 completed the knowledge and attitudes tests. Most (76.5%) caregivers had good knowledge and 45.3% had positive attitudes. Of their daughters, 19.4% had received ≥1 dose of HPV vaccine. Of caregivers of unvaccinated girls, 44.3% and 41.1%, respectively were willing to vaccinate their daughters if vaccination was offered free and at their school. Caregivers of unvaccinated girls were more likely [odds ratio (OR): 3.8] to have been influenced by “other” influences (mainly online articles and anecdotal vaccine injury reports). Of caregivers influenced by their healthcare providers, caregivers of unvaccinated girls were more likely (OR: 0.2) to be influenced by alternative medical practitioners. Caregivers of vaccinated girls were more likely to have good knowledge (OR: 3.6) and positive attitudes (OR: 5.2). Having good knowledge strongly predicted (OR: 2.8) positive attitudes. Having negative attitudes strongly predicted (OR: 0.2) girls being unvaccinated.

**Conclusion:** Providing free school-based HPV vaccination in the private sector may not increase HPV vaccination coverage to an optimal level. Since misinformation was the main driver of negative attitudes resulting in <20% of girls being vaccinated, an advocacy campaign targeting all stakeholders is urgently needed.

## Introduction

In 2018 an estimated 569,847 women globally developed cervical cancer, the third leading cause of cancers affecting women, with 311,365 deaths in 2018 (1, 2). Cervical cancer is the leading cause of cancer in South African women aged 15–44 years, and the overall leading cause of female cancer deaths (2). Although South Africa is home to only 0.73% (20.2 of 2,784.9 million) of the global population of women aged ≥15 years, it bears 2.3% of the global cervical cancer incidence burden, with 12,983 of the 569,847 new cervical cancer cases globally in 2018 being estimated to have occurred in South Africa (1, 2). In addition, poor uptake of cervical cancer screening by South African women results in late presentation (3), thus South African cervical cancer deaths constitute a disproportionately high 1.8% (an estimated 5,595 of 311,365) of estimated global deaths from cervical cancer in 2018 (1, 2).

Persistent infection with high-risk types of human papillomavirus (HPV) has been shown to be the necessary, but not sufficient, cause of cervical cancer (1, 2, 4). Cervical cancer is a vaccine-preventable disease, with three HPV vaccines currently being licensed in many countries throughout the world (4). High-risk HPV types 16 and 18 cause just over 70% of cervical cancer cases globally (1, 2, 4), and are thus targeted by all HPV vaccines, which include quadrivalent, bivalent and nonavalent vaccines, first licensed in 2006, 2007, and 2014, respectively (4). HPV types 6 and 11 are low-risk HPV types that cause about 90% of genital warts, and are targeted by the quadrivalent and nonavalent vaccines (4). The remaining 5 HPV types targeted by the nonavalent vaccine are high-risk types 31, 33, 45, 52, and 58 (4), which together cause 20.1% of global cervical cancers (1). There is also evidence that both the bivalent and quadrivalent vaccines may provide cross-protection against high-risk types 31, 33, and 45 (4), which together cause 12.7% of global cervical cancers (1). The high-risk HPV types targeted by these vaccines also cause varying proportions of cancers of the vulva and vagina; cancers affecting women and men (anal, oropharyngeal, and other head and neck cancers); and penile cancer (1, 2, 4). Thus, these vaccines are all licensed for use in both males and females, and because HPV infection is a sexually transmitted infection, they are best administered before sexual debut, being licensed for use from the age of 9 years (4). Pre-licensure clinical trials and extensive post-marketing surveillance have found that all three vaccines are highly effective and have excellent safety profiles (4).

Despite the excellent safety profile of all HPV vaccines, uptake in many countries has been suboptimal for a variety of reasons, including reasons related to lack of access and vaccine hesitancy (5). One of the key determinants of vaccine hesitancy, defined by the World Health Organization (WHO) as a “delay in acceptance or refusal of vaccination despite availability of vaccination services” (6), is vaccine confidence (6). This concept encompasses several aspects of trust, including trust in the vaccine itself (i.e., that it is safe and effective); trust in the advice and services of the healthcare providers who administer vaccinations; and trust in the national decision-making bodies which mandate or recommend specific vaccines (6). Unfortunately, misinformation spread globally via social media, has eroded public confidence in vaccination, negatively impacting on HPV vaccination uptake in some high-income countries (7).

The bivalent and quadrivalent HPV vaccines have been available in South Africa since 2008 (5). In 2014 the South African National Department of Health, through the Integrated School Health Programme in partnership with the National Department of Basic Education, introduced free HPV vaccination for public sector school girls aged ≥9 years in Grade 4 (5, 8). Two doses of the bivalent HPV vaccine given 6 months apart, are delivered through two annual campaigns in this school-based vaccination programme (5, 8). Data on HPV vaccination coverage in public sector schools are available through the Integrated School Health Programme, with 86.6% coverage of age-eligible girls being reported for the 2014 first dose campaign (8). However, there were some sub-districts with sub-optimal coverage, which may have been related to anti-vaccination messaging on social media during this campaign (8), resulting in vaccine hesitancy (5). Subsequent coverage data have been reported in numbers, not percentages, allowing drop-out rates between dose 1 and 2 to be measured (5). In contrast, data on HPV vaccination coverage in private sector schools, where free HPV vaccination is not offered, are unavailable. This study investigated knowledge, attitudes and practices regarding HPV vaccination, of caregivers of girls aged ≥9 years in grades 4–7 attending private schools in South Africa. Objectives included to (a) determine the level of knowledge that caregivers have about cervical cancer, HPV, and HPV vaccination; (b) describe the attitudes of caregivers toward HPV vaccination; (c) describe the practices of caregivers regarding HPV vaccination; and (d) investigate levels of knowledge and attitudes of caregivers associated with HPV vaccination coverage in girls aged ≥9 years.

## Methods

### Study Design and Study Population

The target population for this cross-sectional survey was caregivers of girls aged ≥9 years in grades 4–7 attending private schools in South Africa in 2018. According to the National Department of Basic Education, there were 1,497 schools attended by 90,722 eligible girls in 2018 (personal communication, Ms Lebogang Phasha, 22 November 2019). Originally, 4 of the 9 provinces were randomly selected (Gauteng, Western Cape, North West, and Limpopo), and all 1,026 schools attended by 66,759 girls in grades 4–7 in those provinces were eligible for inclusion in the survey (see Table 1). Following a poor post-reminder response to the original invitation, a paid Facebook survey-linked advert targeting South African Facebook users aged ≥25 years (caregivers younger than 25 are less likely to have a daughter aged ≥9 years) nationally was run, which effectively extended the study population to include Facebook users from all 9 provinces, who were caregivers of private school girls in grades 4–7.

**Table 1:** Number of private sector schools and girls in grades 4–7 per province in 2018, and study sample.

**Province**	**Private schools**	**Girls in each grade**				**Total girls**	Final sample^**^
		**Grade 4**	**Grade 5**	**Grade 6**	**Grade 7**	***n* (%)**	***n* (%)**
Gauteng^*^	617	12,823	11,403	10,465	9,780	44,471 (49.0)	250 (47.6)
Western Cape^*^	204	2,448	2,283	2,163	1,932	8,826 (9.7)	119 (22.7)
Eastern Cape	166	3,105	2,703	2,589	2,477	10,874 (12.0)	35 (6.7)
Kwa-Zulu Natal	145	1,945	1,824	1,646	1,696	7,111 (7.8)	50 (9.5)
Limpopo^*^	142	2,924	2,638	2,431	2,182	10,175 (11.2)	43 (8.2)
Mpumalanga	80	877	740	655	589	2,861 (3.2)	5 (1.0)
North West^*^	63	959	831	800	697	3,287 (3.6)	15 (2.9)
Free State	58	786	737	604	599	2,726 (3.0)	7 (1.3)
Northern Cape	22	122	98	98	73	391 (0.4)	1 (0.2)
Totals	1,497	25,989	23,257	21,451	20,025	90,722	525

* *Email invitations originally sent to principals of 79.3% (814/1,026) schools with valid email addresses in these 4 provinces*.

** *iOf 615 respondents, 525 reported province of school attended by their daughter/ward*.

### Data Collection Tool and Pre-testing

An online survey was created using the premium version of SurveyMonkey® (https://www.surveymonkey.com/). The survey included 53 items covering socio-demographics (Tables 2, 3), knowledge (Table 4), attitudes (Table 5), and practices (Tables 6, 7) of caregivers regarding HPV vaccination. The tool was pre-tested from 12 January to 21 February 2018 on 6 volunteers, 4 of whom were academics working in the field of vaccine-preventable diseases. The volunteers took an average of 12.16 min to complete the questionnaires, and reported some errors in spelling, grammar, and logical flow. Following editing to correct all errors, the survey was re-tested for a final time on 4 volunteers.

**Table 2:** Frequency distributions of socio-demographics of caregivers, stratified by HPV vaccination status of daughters.

**Caregivers**		**Vaccination status of daughter**			**Total**	
		**Vaccinated**	Unvaccinated^*^/unsure	**Question unanswered**		
		***n* (% vaccinated)**	***n* (% unvaccinated)**	***n* (% unanswered)**	***n* (%)**	**95% CI**
**Relationship with girl (*****n*** **=** **615)**
Biological parents	Mother	67 (84.8)	283 (84.7)	151 (74.8)	501 (81.5)	78.1–84.4
	Father	3 (3.8)	25 (7.5)	13 (6.4)	41 (6.7)	4.9–9.0
Relatives	Grandmother	0 (0.0)	1 (0.3)	10 (5.0)	11 (1.8)	0.9–3.3
	Grandfather	0 (0.0)	1 (0.3)	1 (0.5)	2 (0.3)	0.1–1.3
	Aunt	4 (5.1)	15 (4.5)	16 (7.9)	35 (5.7)	4.1–7.9
	Uncle	2 (2.5)	2 (0.6)	3 (1.5)	7 (1.1)	0.5–2.4
Step parents	Step-mother	0 (0.0)	4 (1.2)	0 (0.0)	4 (0.7)	0.2–1.8
	Step-father	0 (0.0)	1 (0.3)	0 (0.0)	1 (0.2)	0.0–1.1
Legal guardian	Female: 11; Male: 2	3 (3.8)	2 (0.6)	8 (4.0)	13 (2.1)	1.2–3.7
**Age (*****n*** **=** **580)**
	≤ 19	3 (3.8)	15 (4.5)	11 (6.6)	29 (5.0)	3.4–7.2
	20–29	3 (3.8)	8 (2.4)	13 (7.8)	24 (4.1)	2.7–6.2
	30–39	23 (29.1)	112 (33.5)	64 (38.3)	199 (34.3)	30.5–38.4
	40–49	44 (55.7)	180 (53.9)	59 (35.3)	283 (48.8)	44.7–52.9
	50–59	5 (6.3)	17 (5.1)	14 (8.4)	36 (6.2)	4.4–8.6
	≥60	1 (1.3)	2 (0.6)	6 (3.6)	9 (1.6)	0.8–3.0
**Education level (*****n*** **=** **580)**
Graduated with a tertiary qualification:	Diploma	16 (20.3)	75 (22.5)	36 (21.6)	127 (21.9)	18.6–25.5
74.1% (430)	Bachelors degree	17 (21.5)	68 (20.4)	37 (22.2)	122 (21.0)	17.8–24.6
	Honors degree	17 (21.5)	52 (15.6)	23 (13.8)	92 (15.9)	13.0–19.2
	Masters degree	7 (8.9)	37 (11.1)	7 (4.2)	51 (8.8)	6.7–11.5
	Doctoral degree	5 (6.3)	11 (3.3)	5 (3.0)	21 (3.6)	2.3–5.6
	^**^College qualification	1 (1.3)	11 (3.3)	5 (3.0)	17 (2.9)	1.8–4.8
Incomplete tertiary qualification	1 year of college	3 (3.8)	22 (6.6)	7 (4.2)	32 (5.5)	3.9–7.8
	2 years of college	2 (2.5)	11 (3.3)	7 (4.2)	20 (3.4)	2.2–5.4
	3 years of college	0 (0.0)	7 (2.1)	5 (3.0)	12 (2.1)	1.1–3.7
Secondary qualification	12th grade	11 (13.9)	33 (9.9)	24 (14.4)	68 (11.7)	9.3–14.7
Incomplete secondary school education	9th grade	0 (0.0)	2 (0.6)	3 (1.8)	5 (0.9)	0.3–2.1
	10th grade	0 (0.0)	1 (0.3)	3 (1.8)	4 (0.7)	0.2–1.9
	11th grade	0 (0.0)	1 (0.3)	2 (1.2)	3 (0.5)	0.1–1.6
Primary qualification	7th grade	0 (0.0)	1 (0.3)	1 (0.6)	2 (0.3)	0.1–1.4
None/little education	1st grade	0 (0.0)	1 (0.3)	1 (0.6)	2 (0.3)	0.1–1.4
	Did not attend school	0 (0.0)	1 (0.3)	1 (0.6)	2 (0.3)	0.1–1.4
**Employment status (*****n*** **=** **580)**
Employed or self-employed	Full-time employed	47 (59.5)	180 (53.9)	88 (52.7)	315 (54.3)	50.2–58.4
	Self-employed	13 (16.5)	76 (22.8)	34 (20.4)	123 (21.2)	18.0–24.8
	Part-time employed	10 (12.7)	38 (11.4)	13 (7.8)	61 (10.5)	8.2–13.4
Unemployed	Unemployed by choice	4 (5.1)	26 (7.8)	20 (12.0)	50 (8.6)	6.5–11.3
	Seeking employment	5 (6.3)	11 (3.3)	8 (4.8)	24 (4.1)	2.7–6.2
	Retired	0 (0.0)	2 (0.6)	4 (2.4)	6 (1.0)	0.4–2.4
	Disabled; unable to work	0 (0.0)	1 (0.3)	0 (0.0)	1 (0.2)	0.0–1.1
**Race (*****n*** **=** **580)**
	White	54 (68.4)	218 (65.3)	91 (54.5)	363 (62.6)	58.5–66.5
	African	15 (19.0)	57 (17.1)	34 (20.4)	106 (18.3)	15.3–21.7
	Asian/Indian	3 (3.8)	34 (10.2)	21 (12.6)	58 (10.0)	7.7–12.8
	Colored	5 (6.3)	21 (6.3)	19 (11.4)	45 (7.8)	5.8–10.3
	Other	2 (2.5)	4 (1.2)	2 (1.2)	8 (1.4)	0.6–2.8

* *One vaccinated girl changed to unvaccinated for this analysis since her mother did not want her to be vaccinated*.

** *Unspecified*.

**Table 3:** Frequency distribution of socio-demographics of girls (*n* = 479).

**Variable**	***n* (%)**	**95% Cl**
**Age in years**
9	59 (12.3)	9.6–15.7
10	126 (26.3)	22.5–30.5
11	95 (19.8)	16.4–23.8
12	110 (23.0)	19.3–27.1
13	76 (15.9)	12.8–19.5
>13	13 (2.7)	1.5–4.7
**Grade**
4	146 (30.5)	26.4–34.9
5	106 (22.1)	18.5–26.2
6	104 (21.7)	18.2–25.7
7	123 (25.7)	21.9–29.9
**Medical insurance**
Yes	396 (82.7)	78.9–85.9
No	83 (17.3)	14.1–21.1
**Healthcare provider**
Doctor	396 (82.7)	78.9–85.9
Other^*^	39 (8.1)	5.9–11.1
Nurse	29 (6.1)	4.2–8.7
Pharmacist	15 (3.1)	1.8–5.2

* *Homeopath, naturopath, chiropractor, holistic treatment (n = 23)*.

**Table 4:** Caregivers' knowledge about HPV, HPV vaccine, and cervical cancer (*n* = 455).

Statement^*^	**Correct**	**Incorrect**	**Unsure**
	***n* (%)**	***n* (%)**	***n* (%)**
Cervical cancer is a serious disease (True)	435 (95.6)	10 (2.2)	10 (2.2)
HPV vaccination can be obtained in South Africa by consulting a healthcare provider (True)	389 (85.5)	6 (1.3)	60 (13.2)
Cervical cancer is a very rare disease in South Africa (False)	361 (79.3)	18 (4.0)	76 (16.7)
HPV infection can cause cervical cancer (True)	324 (71.2)	42 (9.2)	89 (19.6)
Cervical cancer is one of the most common cancers affecting South African women (True)	315 (69.2)	27 (5.9)	113 (24.8)
Girls should receive HPV vaccination before they become sexually active (True)	276 (60.7)	114 (25.1)	65 (14.3)
The vaccines against cervical cancer are highly effective when given to adult women (False)	228 (50.1)	52 (11.4)	175 (38.5)
The vaccines against cervical cancer prevent 100% of cervical cancers (False)	212 (46.6)	73 (16.0)	170 (37.4)

* The question asked was: Is this statement true or false?

**Table 5:** Caregivers' attitudes toward HPV vaccination (*n* = 455).

Statement^*^	**Strongly disagree**	**Disagree**	**Neutral**	**Agree**	**Strongly agree**
	***n* (%)**	***n* (%)**	***n* (%)**	***n* (%)**	***n* (%)**
Positive statement score	0	1	2	3	4
Allowing my daughter/ward to receive the HPV vaccine will show her that I care about her future health	144 (31.6)	87 (19.1)	59 (13.0)	31 (6.8)	134 (29.5)
Positive statement score	0	1	2	3	4
I want my daughter/ward to be protected against cervical cancer by being vaccinated against HPV	129 (28.4)	32 (7.0)	54 (11.9)	115 (25.3)	125 (27.5)
Positive statement score	0	1	2	3	4
I believe that the HPV vaccine is safe and effective for the prevention of cervical cancer	127 (27.9)	32 (7.0)	92 (20.2)	128 (28.1)	76 (16.7)
Positive statement score	0	1	2	3	4
I think that it is important for young girls to be vaccinated against cervical cancer	127 (27.9)	33 (7.3)	51 (11.2)	114 (25.1)	130 (28.6)
Negative statement score	4	3	2	1	0
It worries me that children receive so many vaccines these days	75 (16.5)	107 (23.5)	73 (16.0)	78 (17.1)	122 (26.8)
Negative statement score	4	3	2	1	0
I am worried about the rumors regarding the side-effects of the HPV vaccine	36 (7.9)	60 (13.2)	100 (22.0)	101 (22.2)	158 (34.7)

* *To what extent do you agree with each of the following statements? Please indicate your response by selecting the appropriate box using the following scale: strongly disagree, disagree, neither agree nor disagree, agree, strongly agree*.

**Table 6:** Practices of caregivers of vaccinated girls (*n* = 80).

**Variable**	***n* (%)**	**95% CIs**
**Doses received (*****n*** **=** **80)**
1	31 (38.8)	28.1–50.3
2	34 (42.5)	31.5–54.1
3	15 (18.8)	10.9–29.0
**Type of vaccine (*****n*** **=** **80)**
Bivalent	15 (18.8)	10.9–29.0
Quadrivalent	28 (35.0)	24.7–46.5
Unsure^*^	37 (46.3)	35.0–57.8
**Who/where vaccinated (*****n*** **=** **80)**
Doctor	17 (21.3)	12.9–31.8
Healthcare clinic	21 (26.3)	17.0–37.3
Pharmacist	10 (12.5)	6.2–21.8
Other^**^	32 (40.0)	29.2–51.6
**Medical insurance paid (*****n*** **=** **80)**
No^**^	64 (80.0)	69.6–88.1
Yes	16 (20.0)	11.9–30.4
**Access to HPV vaccination information (*****n*** **=** **80)**
Yes	58 (72.5)	61.4–81.9
No	18 (22.5)	13.9–33.2
Not sure	4 (5.0)	1.4–12.3
**Main influencer of decision to vaccinate (*****n*** **=** **79)**
Allopathic healthcare provider	57 (72.2)	60.9–81.7
Other^***^	16 (20.3)	Not available
School principal/class teacher	4 (5.1)	1.4–12.5
Alternative medical practitioner	2 (2.5)	0.3–8.9

* *26 of “unsure” vaccinated at school thus received the bivalent vaccine*.

** *31 vaccinated at school for free (1 without parent's consent); 1 vaccinated by parent at home*.

*** *Includes unanalysed free text data*.

**Table 7:** Practices of caregivers of unvaccinated girls (*n* = 312).

**Variable**	***n* (%)**	**95% CIs**
**Willing to vaccinate if vaccine provided free (*****n*** **=** **305)**		
Yes	135 (44.3)	38.6–50.0
No	130 (42.6)	37.0–48.4
Unsure	40 (13.1)	9.6–17.6
**Willing to vaccinate if vaccine provided at school (*****n*** **=** **304)**		
No	145 (47.7)	42.0–53.5
Yes	125 (41.1)	35.6–46.9
Unsure	34 (11.2)	8.0–15.4
**Access to information about HPV vaccine (*****n*** **=** **308)**		
Yes	176 (57.1)	51.4–62.7
No	97 (31.5)	26.4–37.1
Unsure	35 (11.4)	8.2–15.6
**Main influencer of decision to not vaccinate (*****n*** **=** **311)**		
Other^*^	153 (49.1)	Not available
Allopathic healthcare provider	131 (42.1)	36.6–47.8
Alternative medical practioner	23 (7.4)	4.9–11.0
School principal/class teacher	4 (1.3)	0.4–3.5

* *Includes unanalysed free text data*.

### Data Collection via Emails to School Principals

Of all 1,026 private schools offering tuition to girls in grades 4–7, email addresses for 904 were provided by the National Department of Basic Education. Invitations to participate were emailed to the school principals of these 904 schools during March 2018. For emails that bounced back, valid email addresses for schools were searched for electronically using Google, and followed up telephonically where telephone numbers were available. Emails were resent to all valid addresses during May and June 2018. Reminder emails were sent to all valid email addresses during August and September 2018. Ethics clearance (TREC/289/2017/PG) for this study was obtained from the University of Limpopo Turfloop Research Ethics Committee, and a copy of the clearance certificate was attached to all emails.

Each email contained two separate invitations, one addressed to the school principals, and one to the caregivers. The invitation to the principals explained the rationale for the study, and requested principals to email the invitation to the relevant caregivers. To increase the response rate, principals were informed that all school names submitted by participants in the survey, would be entered into a lucky draw to win R20,000 in gift vouchers. The invitation to the caregivers provided information on the aim and objectives of the study followed by a consent statement and an option to accept or decline participation in the study, with a link to the online survey if they consented to participate. Caregivers were informed about the lucky draw, and assured that the survey was completely voluntary and anonymous, with the only personal information requested being the name of their daughter's school for entry into the lucky draw. Those who clicked on the “accept” hyperlink were directed to the anonymous questionnaire. Participants were requested to select the most applicable answers from the drop-down lists provided for each question. Free text answers were limited to questions where the option “other (please specify)” was selected.

### Data Collection via Facebook Advert

A paid Facebook advert was run for 4 days from 31 October to 3 November 2018. In addition, the advert was placed on the South African Vaccination and Immunisation Centre's Facebook page (https://www.facebook.com/savicinfo/) for 20 days from 31 October to 19 November 2018. The advert was worded as follows: “If you have a daughter in grades 4–7 attending a South African private school, please click on the link below to participate in an anonymous HPV vaccination survey, and stand a chance for your daughter's school to win R20,000 in gift vouchers.” Clicking on the link took users to the same information contained in the email invitation to caregivers, with a link to the anonymous survey for those consenting to participate.

### Data Analysis

#### Knowledge of Caregivers

There were 8 statements included in the knowledge test (Table 4), and respondents had to select one option from the following: “True,” “False,” “Unsure.” Each correct answer was scored 1, while incorrect answers (including “Unsure”) were scored 0. The total possible score was thus 8. The mean and median scores, score range and standard deviation (SD) were calculated. Furthermore, knowledge scores were converted to categorical data using cut-offs. Knowledge categories were poor (score: 0–3), average (score: 4), and good (score: 5–8).

#### Attitudes of Caregivers

There were 6 statements included in the attitude test (Table 5), and respondents had to select one option from a 5-point Likert scale: “Strongly disagree,” “Disagree,” “Neither agree nor disagree,” “Agree,” “Strongly agree.” Positive answers were scored from 4 to 0 for each respective option, while negative statements were scored from 0 to 4. The possible scores thus ranged from 0 to 24. The mean and median scores, score range and SD were calculated. Furthermore, attitude scores were converted to categorical data using cut-offs. Attitude categories were negative (score: 0–11), neutral (score: 12) and positive (score: 13–24).

#### HPV Vaccination-Related Practices of Caregivers

Categorical data were collected on vaccination status, doses received, vaccine type, where vaccination was received, medical insurance coverage, access to information and main influencer of vaccination decision (Table 6). Caregivers of unvaccinated girls were also asked about their willingness to have their daughters vaccinated if vaccination was free and provided through their school (Table 7). Frequency distributions using percentages and 95% confidence intervals (95% CI) were calculated.

#### Associations Between Knowledge and Practice, and Attitudes and Practice

Knowledge scores were re-categorised into good knowledge (scores ≥5) and average/poor knowledge (scores ≤ 4), and the attitude scores were re-categorised into positive attitudes (scores ≥13) and neutral/negative (scores ≤ 12). Inferential data analysis was used to measure associations between knowledge and HPV vaccination coverage, and attitudes and HPV vaccination coverage, and their statistical significance. These statistics included odds ratios (ORs), the 95% CI around the ORs, and chi-square *p*-values for the ORs (Table 8). Statistical significance was set at *p* < 0.05.

**Table 8:** Associations between vaccination status and knowledge and attitudes of caregivers.

		**Ever received HPV vaccine**				
		**Yes**	No^*^	**OR**	**95% CI**	***p*-value**
		***n* (%)**	***n* (%)**			
Knowledge	Good	72 (91.1)	248 (74.3)	3.6	1.6-8.0	0.001
	Average/poor	7 (8.9)	86 (25.7)			
Attitude	Positive	61 (77.2)	132 (39.5)	5.2	2.9-9.2	0.000
	Neutral/negative	18 (22.8)	202 (60.5)			
	Totals	79^**^	334^**^			

* *“No” and “unsure” were added together for this analysis*.

** *One vaccinated girl changed to unvaccinated for this analysis since her mother did not want her to be vaccinated*.

## Results

### Response Rates

#### Response From Email Invitations Sent via School Principals

Of the 904 schools with email addresses on the National Department of Basic Education list, 814 had valid email addresses. Thus, only 79.3% (814/1,026) of schools could be reached. Since the National Department of Basic Education list did not contain a breakdown of numbers of girls per school, it was assumed that ~79.3% of 66,759 (i.e., 52,940) of caregivers may have been reached if all principals forwarded the invitation to all caregivers of eligible girls. On 25 March 2018, the first author received an email from a director of one of the school groups, alleging that the information the survey was collecting, was confidential, and requesting further information regarding the purpose of the study, university approval and government mandate for such research. A reply was sent by the corresponding author (supervisor of the first author), reiterating the information supplied in the original invitation, namely (a) the total anonymity of the online survey with no personal identification data being captured; (b) the purpose of the study; and (c) the approval by the university, with the ethics clearance certificate again being attached. In addition, an explanation was added regarding the research being in line with the National Department of Health's Strategic Plan 2014–2019 (with the plan attached, and reference made to the HPV vaccination first dose national coverage target of 90% for 2018/2019), and being funded by the National Research Foundation of South Africa, which only funds research that is in line with national priorities. On 27 March a reply from the school group was received, stating: “Thank you for considering (name of school group) for this research—however, it is a policy of ours not to participate in research projects in order to protect our brand and learners.” The supervisor replied with an explanation of the ethical implications of (a) blocking research that has public health benefits; and (b) the directors' assumption that parents are unable of making their own informed decisions, whereas autonomous decision-making is a human right, protected by South Africa's constitution (https://www.gov.za/documents/constitution-republic-south-africa-1996). Of 814 schools emailed between 19 March and 16 June, 2.0% (16/814) were named by 139 respondents as the school their daughter was attending. There were 139 responses by 14 August, the day before the reminder emails were sent out. After sending the reminder emails between 15 August and 27 September 2018, by 30 October 2018, the day before the Facebook advert was placed, the proportion of schools named by respondents increased to 2.3% (19/814), while responses had increased to 167. The 13 schools belonging to the school group declining to participate were not named by any respondents. Since 2.3% of the schools had responded, the approximate number of girls whose caregivers received the invitation to participate was calculated as 2.3% of 52,940 (see Table 1 for totals of girls in each of the 4 provinces), giving 1,218. This gave a response rate of only 13.7% (167/1,218).

#### Response From Facebook Advert

The paid Facebook advert placed from 31 October to 3 November 2018 had the potential (since it is unlikely that all caregivers were Facebook users, and even if they were, it is unlikely that all Facebook-using caregivers accessed Facebook during this time) to reach all caregivers of the 90,722 eligible girls, since it targeted all South African Facebook users aged ≥25 years from all 9 provinces. By the time the advert was withdrawn on 3 November, it had been viewed by 118,105 Facebook users (views are called “Impressions” by Facebook), and the responses had increased by 395 to 562. The number of caregivers of eligible girls who were among those who viewed the advert is unknown. Also, the number of caregivers of eligible girls who use Facebook is unknown. It is thus not possible to calculate a response rate for this increase of 395. In addition, the free advert that was placed on the South African Vaccination and Immunisation Centre's Facebook page from 31 October to 19 November 2018 attracted several anti-vaccination responses within the first 2 days, calling for the survey to be boycotted, with links to HPV vaccine misinformation posted on the Vaccine Awareness South Africa Facebook page (https://www.facebook.com/groups/vaccineawarenessvasa). On 2 November these anti-vaccination posts were deleted, and the ability to reply to SAVICinfo posts was blocked by the Facebook page administrator. This free advert brought in an additional 53 responses after 3 November.

#### Total Response

In total 615 responses (448 post-Facebook advert) were received, with 615 respondents completing the question on their relationship to the girl; 580 completing the socio-demographic section; 479 completing the section on health insurance coverage, age and grade of the girl; 455 completing the knowledge and attitudes tests; and 413 providing data on the HPV vaccination status of their daughters (or wards in the case of legal guardians; daughters will be used hereafter for daughters or wards). When comparing respondents who completed the knowledge and attitude tests to those who did not complete them, three statistically significant differences (p ≤ 0.05) were found. Those who completed the tests were older (59.6% vs. 45.6% were ≥40 years); more educated (86.8% vs. 79.2% had at least some tertiary education); and more likely to be the biological parent of the girl (90.3% vs. 81.9%), than respondents who did not complete these tests. Respondents spent on average 11.04 min to complete the questionnaire. Of 525 caregivers answering the question on name of school, 508 gave valid school names, naming 301 schools attended by 500 girls, with 8 girls being homeschooled. Thus, in total, 37.0% (301/814) of schools were reached. Using the database numbers and randomizer.org, one of these schools was randomly selected for the lucky draw. This school was one of those receiving the emailed invitation, but had not been named in the responses received before placing the Facebook advert. The school was contacted via email, and after no response was received an alternative email address and telephone number were found on the internet. The original and alternative email addresses were confirmed telephonically, and an email was sent to both email addresses. No response was received.

### Socio-Demographics

Of the caregivers, 86.4% (501/580) were South African by birth, and 91.4% (562/615) were female, with 88.1% (542/615) being biological parents of the girls. See Table 2 for further details. While girls from all nine South African provinces were represented in the final sample, 70.3% (369/525) attended private schools in Gauteng and Western Cape provinces. See the last column of Table 1 (Final sample) for details on provinces of schools, and Table 3 for details on socio-demographics of girls.

### Caregivers' Knowledge About Cervical Cancer, HPV, and HPV Vaccination (*n* = 455)

**Figure 1:**
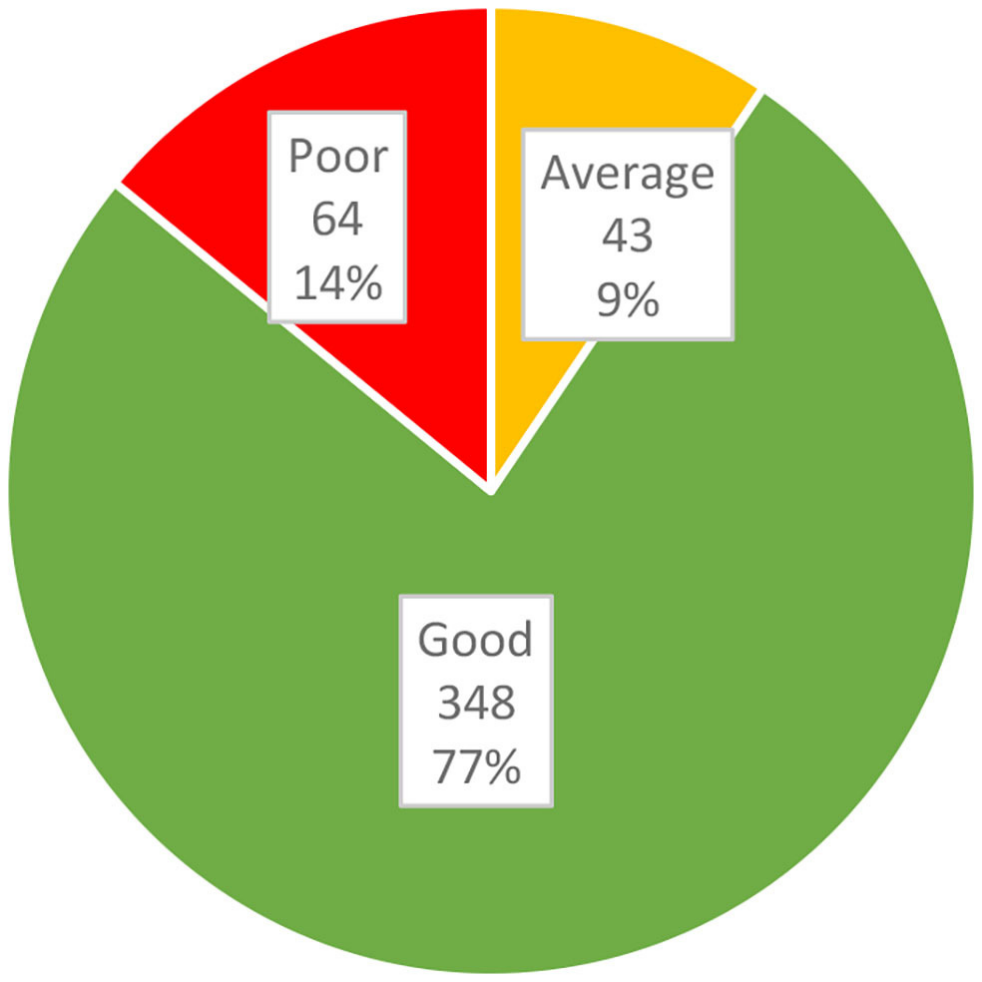
Categories of caregivers' knowledge (*n* = 455).

Knowledge scores ranged from 0/8 obtained by 0.7% (3/455) of respondents, to 8/8 obtained by 10.3% (47/455) of respondents, with a mean of 5.6 (SD: 1.7) and a median of 6.0. Figure 1 illustrates the proportions of knowledge scores collapsed into categorical data. The frequency distribution of answers to the knowledge test is shown in Table 4.

### Caregivers' Attitudes Toward HPV Vaccination (*n* = 455)

**Figure 2:**
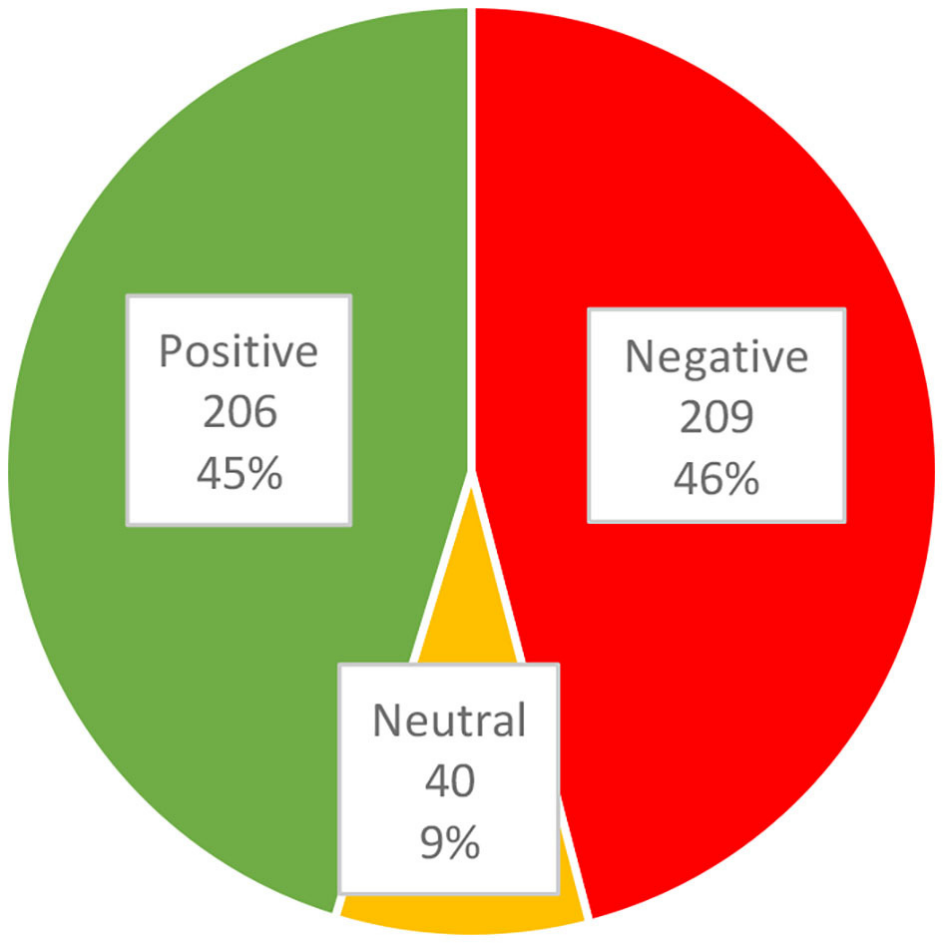
Categories of caregivers' attitudes (*n* = 455).

Attitude scores ranged from 0/24 obtained by 3.1% (14/455) of respondents, to 24/24 obtained by 0.2% (1/455) of respondents, with a mean of 11.4 (SD: 5.3) and a median of 12.0. Figure 2 illustrates the proportions of attitude scores collapsed into categorical data. The frequency distribution of answers to the attitude test is shown in Table 5.

### Caregivers' Practices Regarding HPV Vaccination of the Girls (*n* = 413)

Of the daughters of 413 respondents who answered this section, 19.4% (80/413) had received ≥1 dose of HPV vaccine. Healthcare facilities had been used for 60.0% (48/80) of the vaccinations, while 1.3% (1/80) of the caregivers had obtained the non-avalent vaccine from overseas and vaccinated her daughter, and 38.8% (31/80) reported that their daughter was vaccinated at school. Of girls who were vaccinated in healthcare facilities, 87.5% (42/48) had medical insurance, and of these 38.1% (16/42) of vaccinations were covered partially or in full by medical insurance. Of girls who were vaccinated at school, 19.4% (6/31) of caregivers clarified that the school was the previous public sector school attended by their daughter, and of these, one caregiver added that parental consent was not obtained, and that she was opposed to vaccination. Since school-based HPV vaccination is confined to public-sector schools in South Africa, all 31 girls who received vaccination at their schools must have been in public schools at that time, and were thus vaccinated free of charge. Of caregivers whose daughters were vaccinated, 41% (33/80) paid the full cost of the vaccine. The main influence on their decision to vaccinate, cited by 74.7% (59/79) of caregivers, was advice given by a healthcare provider (57 allopathic practitioners and 2 alternative medical practitioners). See Table 6 for further details.

The remaining 80.6% (333/413) of girls were either not vaccinated [75.5% (312/413)], or the respondents were unsure about their vaccination status [5.1% (21/413)]. The main influence on their decision to not vaccinate, cited by 49.5% (154/311) of caregivers of unvaccinated girls, was advice given by a healthcare provider (131 allopathic practitioners and 23 alternative medical practitioners). Of caregivers of unvaccinated girls who answered both questions on willingness to vaccinate, 39.8% (121/304) were willing if vaccinations were offered for free at their daughter's school. See Table 7 for further details.

When comparing vaccinated to unvaccinated girls, there were statistically significant differences in some caregiver practices. Caregivers of vaccinated girls were (a) twice as likely to have access to HPV vaccination information (OR: 2.0; 95% CI: 1.2–3.4; *p* = 0.006); and (b) almost four times more likely to have based their vaccination decision on the advice of their allopathic healthcare provider (OR:3.6; 95% CI: 2.1–6.1; *p* = 0.000). In contrast, caregivers of unvaccinated girls were almost four times more likely to have based their vaccination decision on “other” influences (OR: 3.8; 95% CI: 2.1–6.9; *p* = 0.000). While these responses were written in “free text” and coding is still underway, thus far, these “other” influences are mainly online articles and anecdotal reports of vaccine injuries. In addition, of caregivers influenced by advice from their healthcare providers (either allopathic or alternative), caregivers of unvaccinated girls were five times more likely to be influenced by advice from alternative medical practitioners (OR: 0.2; 95% CI: 0.05–0.9; *p* = 0.007). Although there were some socio-demographic differences between caregivers of vaccinated and unvaccinated girls (see Table 2), none of these were statistically significant. There was also no statistically significant difference in medical insurance coverage, with 82.3% (65/79) of consenting vaccinated girls and 83.8% (280/334) of girls whose caregivers were unsure or had not consented (including 1 girl who was vaccinated without consent), having medical insurance.

### Association Between Girls' HPV Vaccination Status and Their Caregivers' (a) Knowledge and (b) Attitudes

Of the 455 respondents who completed the knowledge and attitude tests, 413 had answered the question on whether their daughter had received any HPV vaccinations. Since one of the parents had indicated that she was totally opposed to vaccination, and her daughter had received one dose of HPV vaccine without parental consent at the public sector school she had previously attended, the vaccination status for this record was changed to unvaccinated for the two association analyses. There were statistically significant associations between knowledge, attitudes and uptake of the HPV vaccine. Caregivers of vaccinated girls were almost four times more likely to have good knowledge scores, and five times more likely to have positive attitude scores. See Table 8 for further details. Conversely, caregivers' negative attitude toward HPV vaccination strongly predicted their daughters being unvaccinated (OR: 0.2; 95% CI: 0.1–0.3; *p* = 0.000). In addition, caregivers with good knowledge scores were almost three times more likely to have positive attitudes than those with poor knowledge scores (OR: 2.8; 95% CI: 1.7–4.7; *p* = 0.000).

## Discussion and Conclusion

### Response Rate

Despite attempts to improve response rates, such as offering a prize, online surveys generally achieve lower response rates than paper-based surveys (9). The poor response rate of 10.9% from the email invitation sent via school principals was similar to the 9% response rate to an online cross-sectional survey on HPV vaccination acceptability among non-medical academics working at the University of KwaZulu-Natal (10). However, the first attempt to conduct this survey of caregivers via email, was possibly hampered by school principals not forwarding the email on to parents. Other than the refusal by one school group which effectively prohibited caregivers of girls from 13 schools from participating, this supposition is further supported by the proportion of named schools increasing from 2.0% for the emailed invitation-based survey, limited to 4 provinces, to 37% of schools nationally after placing the Facebook advert, with the vast majority of responses in the final sample being from the 4 provinces originally targeted by the email invitation. Also noteworthy is the non-response from the principal of the school that won the lucky draw prize, which suggests that this school, and possibly the school group to which this school belongs, may also not have participated in forwarding the email invitation to caregivers. In addition, 167 responses were collected in 7 months in response to the email invitation and reminders, while 395 were collected in just over 3 weeks through Facebook. This is evidence that social networking sites are an effective platform for conducting health surveys in South Africa, as in other countries (11). However, the call from anti-vaccination lobbyists to boycott the survey may have had a negative impact on the responses rate. Finally, the finding that respondents who completed the knowledge and attitudes tests were older and more educated than those who did not, suggests that pretesting on volunteers who were predominantly academics may have resulted in these tools being overly complicated, or unappealing to younger respondents.

### Caregivers' Knowledge About HPV, HPV Vaccination, and Cervical Cancer

Low levels of knowledge about the link between HPV infection and cervical cancer is an important barrier to HPV vaccination uptake. A review of 27 studies conducted in sub-Saharan African countries, including South Africa, before national HPV vaccination programmes were introduced, found low levels of knowledge in all 16 studies investigating knowledge of cervical cancer, HPV, and HPV vaccine (12). In contrast, 76.5% of respondents in this study had good knowledge about HPV, HPV vaccination, and cervical cancer. Given that 74% of respondents had a tertiary education, this finding was not unexpected, since having a college education was found to be a statistically significant predictor of American adults being knowledgeable about HPV, HPV-associated cancers, and the HPV vaccine (13). This finding has also been echoed by a South African study conducted on women utilising public sector health services in KwaZulu-Natal in 2018, where having tertiary education was statistically significantly associated with higher levels of knowledge and awareness about HPV and HPV vaccination (14). Most published South African studies have focused on measuring awareness instead of knowledge, with, for example, very high levels of awareness found in non-medical academics in KwaZulu-Natal, a few months before the national roll-out of the first dose of HPV vaccination in 2014 (10). Those that have measured knowledge in the general female population have found very low levels of knowledge in less educated populations before national HPV vaccination introduction (5, 12), and after national introduction (15). For example, 98% of women attending clinics in a rural part of Limpopo Province who were surveyed in 2015, a year after the national roll-out, had very low levels of knowledge about HPV and HPV vaccination (15).

### Caregivers' Attitude Toward HPV Vaccination

Positive attitudes of caregivers toward HPV vaccination is a strong predictor of HPV vaccination uptake by their children, thus effective interventions to improve attitudes are considered vital for increasing HPV vaccination uptake (16). Acceptability of HPV vaccination is often measured instead of attitudes and practices, especially when the vaccine has not yet been incorporated into the national programme (12, 17), with high levels of acceptability being interpreted as a positive attitude toward the vaccine. All 12 studies investigating acceptability levels of HPV vaccination in sub-Saharan African countries, including South Africa, included in a systematic review conducted in 2014, reported high levels of acceptability of HPV vaccination (12). In addition, a survey of non-medical academics in KwaZulu-Natal in 2013–2014, found high acceptability levels that increased from a baseline of 79–88% after exposure to a fact-sheet on cervical cancer, HPV transmission, and vaccination (10). Also, the KwaZulu-Natal study conducted on 200 women utilizing public sector health services in 2018, found that almost all participants (only one participant was unsure) would encourage others to use the HPV vaccine, demonstrating a positive attitude across all levels of education, although all participants also said they would first need more information, with 75% being unsure of the safety of the vaccine (14). In contrast, the study conducted on rural Limpopo Province women in 2015 which found very low levels of knowledge, also found that 92% of women had negative attitudes toward the vaccine (15). In the current study, 45.9% of caregivers had a negative attitude toward HPV vaccination, with more than 50% (a) agreeing or strongly agreeing that they are concerned about the rumors of HPV vaccine side effects, and (b) disagreeing or strongly disagreeing that vaccinating their daughter would demonstrate that they care about her future health. This finding was not unexpected, given the increasing presence of anti-vaccination lobbying on the South African internet (18), negative social media messaging specifically targeting the HPV vaccine before the vaccine was introduced in 2014 (8), and continual post-introduction negative media coverage of the HPV vaccine, both in the South African mainstream news media (for example https://www.iol.co.za/news/south-africa/western-cape/cervical-cancer-vaccine-gave-my-child-brain-disease-8791732) and on South African social media (19).

### Practices of Caregivers Regarding HPV Vaccination

In April 2020, the WHO published a draft “Global strategy toward eliminating cervical cancer as a public health problem,” with one of the goals being that by 2030, in all member countries 90% of girls aged 15 years should be fully vaccinated with the HPV vaccine (20). While South Africa's public sector HPV vaccination programme does not seem to be on track to reach this goal (5), the situation in the private sector appears to be far more concerning, with this study finding that only 19.4% of age-eligible girls attending South African private schools had received one or more doses of HPV vaccine.

A systematic review of strategies to address vaccine hesitancy, found that one of the most effective strategies for increasing vaccination uptake, were those aimed at improving convenience and access to vaccination (21). An important access factor that may negatively impact HPV vaccination coverage in the private sector, is cost (5). Of the caregivers of vaccinated girls in this study, 41% had paid the full cost of the vaccine, with medical insurance partially or fully covering the cost of 20%, while 39% did not pay as the girls received free vaccination through their previous public sector school. Cost does indeed seem to have negatively impacted vaccination uptake, but not to a very large extent, since only 44% of caregivers of unvaccinated girls were willing to have their daughters vaccinated if HPV vaccination was provided free of charge. Another access factor that must be considered is inconvenience, since HPV vaccination is not offered at South African private sector schools, thus girls need to be transported to private health facilities to be vaccinated. Since only 41% of caregivers of unvaccinated girls were willing to have their daughters vaccinated if it was offered at school, the negative impact of inconvenience was not as large as expected. Thus, while access would be greatly improved by providing free HPV vaccination at private sector schools, it seems unlikely that this alone would bring first dose vaccination coverage up to 86.6% as experienced in the public sector in 2014 (8), and almost certainly not up to the WHO target of 90% by 2030 (20).

The need for broad-based, widely accessible communication and social mobilisation to increase the uptake of HPV vaccination in South Africa, has recently been emphasised (5). This communication must be evidence-based, which is the case for caregivers of girls attending public sector schools. These caregivers have access to evidence-based HPV vaccination information, regardless of whether they are actively seeking such information or not, through the HPV vaccination programme. In contrast, if caregivers of private school girls want HPV vaccination information, they must obtain it elsewhere, with no guarantee that the information they obtain is evidence-based. As can be seen from the results of this study, caregivers of vaccinated girls were twice as likely to have access to HPV vaccination information, with 72% reporting such access. While 57% of caregivers of unvaccinated girls reported they had access to information, the quality of this ‘information' is clearly questionable, since this “information” led them to abstain from vaccinating their daughters.

An important source of HPV vaccination information is advice given by healthcare providers (5). The systematic review of strategies to address vaccine hesitancy mentioned previously, found that other most effective strategies for increasing vaccination uptake, were those aimed at healthcare providers (21). This is because the vaccination-related advice given by healthcare providers is well-established as one of the most important factors in caregivers' HPV vaccination decision-making (5). This was found to be true for 75% and 50% of caregivers of vaccinated and unvaccinated girls, respectively in this study. However, caregivers of vaccinated girls were four times more likely to have been influenced by advice from their allopathic healthcare providers, and of all caregivers whose vaccination decisions were influenced by advice from their healthcare providers (either allopathic or alternative), caregivers of unvaccinated girls were five times more likely to be influenced by advice from alternative medical practitioners. This finding is supported by a study on South African internet-based anti-vaccination lobbying, which found that the majority of healthcare providers who authored internet-based anti-vaccination articles were complementary or alternative medical practitioners (18).

While the internet is an excellent source of HPV vaccination information, it is also an important source of HPV vaccination misinformation for South African internet users seeking information to help them make vaccination-related decisions (18). Even when not seeking this information, South African social media users are being exposed to this misinformation, which may negatively impact or change their decisions, causing them to refuse HPV vaccination for their daughters (5, 8, 19). This study found that caregivers of unvaccinated girls were almost four times more likely to have based their decisions on “other” influences, including online articles and anecdotal reports of vaccine injuries. This finding highlights the importance of providing all caregivers, not only those with daughters in public sector schools, with evidence-based information, which may to a large extent curtail the need for private sector caregivers to seek this information on the internet, and limit their exposure to (and belief of) social media-based misinformation.

### Levels of Knowledge and Attitudes Associated With HPV Vaccination Coverage

Interventions focused on building high levels of awareness and knowledge about vaccination, and positive attitudes toward vaccination, are important for addressing vaccine hesitancy and increasing vaccination uptake (16, 21). In sub-Saharan Africa, a positive attitude toward HPV vaccination seems to be a much stronger predictor of vaccine acceptability/acceptance than having high knowledge levels (12). This assumption is based on the finding that despite low levels of knowledge, all 12 studies investigating acceptability levels of HPV vaccine included in a systematic review conducted in 2014, before HPV vaccination was rolled out nationally, reported high levels of acceptability of HPV vaccination (12). However, levels of acceptability of a vaccine that is not yet available, may not translate directly into the same levels of uptake of the vaccine once it becomes available, especially when social media-based anti-vaccination lobbying has eroded public confidence in the interim. This study, conducted 4 years after the national rollout of South Africa's public school-based HPV vaccination programme, found that caregivers of vaccinated girls were almost four times more likely to have good knowledge scores, and five times more likely to have positive attitude scores. Also, caregivers with good knowledge scores were almost three times more likely to have positive attitudes than those with poor knowledge scores. So while having a positive attitude was a more important driver of vaccination uptake, having good knowledge was also associated with having a positive attitude. This again emphasises the importance of providing evidence-based HPV vaccination information to caregivers of private sector school girls, to improve both their knowledge and their attitudes. Furthermore, the systematic review of strategies to address vaccine hesitancy, found that other most effective strategies for increasing vaccination uptake, were those involving increasing vaccination knowledge and awareness (21).

### Study Limitations

Since online surveys can only be accessed by those with internet access, and emailed invitations can only be sent to those with email accounts, this study suffers from selection (sampling) bias. However, private schooling in South Africa is costly, and it is likely that the majority of caregivers who can afford to send their children to private schools, will also afford to have internet access, and have email addresses, thus the impact of this bias may be minimal. However, caregivers without Facebook accounts were excluded from the Facebook advert phases of the study, which introduces further selection bias. Because those who use social media are more likely to be exposed to vaccination misinformation and anti-vaccination lobbying (7), this bias may have resulted in an over-representation of caregivers who were vaccine hesitant, with a subsequent under-estimation of HPV vaccination uptake and positive attitudes. On the other hand, the refusal of some school principals to participate in the email phase of the study, and the call from anti-vaccination lobbyists to boycott the Facebook advert phase of the survey, has introduced another type of selection bias (non-response/volunteer bias), which may have resulted in an under-representation of caregivers with negative attitudes and practices regarding HPV vaccination. This may have resulted in an over-estimation of HPV vaccination uptake and positive attitudes.

In addition, unlike the emailed invitation phase of the study, there was no way of verifying that those participating in the Facebook advert phase of the study were in fact caregivers of age-eligible girls attending private schools in South Africa. For example, 29 of the caregivers were aged ≤ 19, which in most high-income countries may be considered far too young for being a caregiver. However, the reality in South Africa, as in many other African countries where human immunodeficiency virus infection is highly endemic, is that unfortunately, there are many child-headed households (22). In addition, many South African private schools and non-governmental organizations offer scholarships and bursaries for disadvantaged children (https://www.advance-africa.com/bursaries-for-private-schools-in-south-africa.html). Taken together with possible selection bias, the findings of this study may not be generalisable to all South African caregivers of age-eligible girls attending private schools.

### Conclusion and Recommendations

To the best of our knowledge, this is the first report on HPV vaccination in South African private sector schools. The finding that <20% of girls had received HPV vaccination, despite the majority of caregivers having good knowledge regarding HPV, cervical cancer and HPV vaccination, is highly concerning. While access issues (i.e., cost of vaccination and inconvenience of using health facility-based services) were clearly problems for many caregivers, solving these problems by providing free school-based HPV vaccination programmes, has the potential to increase HPV vaccination uptake by only 40%. Misinformation appears to be the main driver of negative attitudes resulting in low vaccination uptake, with caregivers of unvaccinated girls being influenced by online articles and anecdotal reports of vaccine injuries, and advised by misinformed healthcare providers, school principals and teachers.

Given the limitations of this study, private sector HPV vaccination coverage needs to be further investigated. Since data sources such as medical insurance companies (23) and private sector vaccination providers (24) have been utilised to provide coverage statistics in the United States of America (USA), these sources could possibly be utilised in a follow-up study to validate the very low coverage reported in this study.

While further research is required in order to understand the complex barriers preventing HPV vaccination acceptance among caregivers (5), the results of this study can be used to inform future policies. The call for extending free HPV vaccination to private sector schools (5) is strengthened by these results; however, they also suggest that optimal herd immunity against cervical cancer may not be achieved through a free fully inclusive national school-based HPV vaccination programme. There is an urgent need to build confidence in HPV vaccination, including and especially amongst healthcare providers, as they are important influencers of caregivers' vaccination decisions. Given the importance of the role that advice from misinformed healthcare providers played in the low vaccination uptake found in this study, education of healthcare providers is vital, and has been shown to be one of the best interventions in addressing vaccine hesitancy and increasing vaccination uptake (21). A global review of strategies to address vaccine hesitancy found that dialogue-based interventions, including via social media and mass media, were among the most promising (21). In the USA, interventions utilising communication technologies (including interactive computer videos; electronic health record prompts; automated reminders via text messages, e-mail, telephone call or Facebook) were reported to increase HPV vaccination uptake, with most interventions targeting parents being successful (25). All of these interventions are feasible in South Africa, where currently HPV vaccine advocacy is confined to public sector school campaigns. Thus, additional HPV vaccination advocacy campaigns are needed, and these should be directed at all stakeholders—policy makers, the media, educators, healthcare providers, and the general public, including adolescents.

## Data Availability Statement

The raw data supporting the conclusions of this article will be made available by the authors, without undue reservation.

## Ethics Statement

The study involving human participants was reviewed and approved by the University of Limpopo Turfloop Research Ethics Committee. The participants accepted a consent statement to participate in this study.

## Author Contributions

RB is the grant holder for the over-arching project under which this study falls, and is the supervisor of TM. JM and CD are co-investigators in the over-arching project, and together with RB developed the grant proposal, and took part in pre-testing and refining the data collection tool. TM wrote the research proposal and obtained ethics clearance, and conducted data collection, data analysis and dissertation writing under the supervision of RB. All authors gave input into the manuscript, and take equal responsibility for the contents of the manuscript.

## Conflict of Interest

The South African Vaccination and Immunisation Centre, and the Network for Education and Support in Immunisation, receive unrestricted educational grants from the vaccine industry.
